# Hepatoma-Derived Growth Factor-Related Protein-3 Is a Novel Angiogenic Factor

**DOI:** 10.1371/journal.pone.0127904

**Published:** 2015-05-21

**Authors:** Michelle E. LeBlanc, Weiwen Wang, Nora B. Caberoy, Xiuping Chen, Feiye Guo, Gabriela Alvarado, Chen Shen, Feng Wang, Hui Wang, Rui Chen, Zhao-Jun Liu, Keith Webster, Wei Li

**Affiliations:** 1 Bascom Palmer Eye Institute, Department of Ophthalmology, University of Miami School of Medicine, Miami, Florida, United States of America; 2 Vascular Biology Institute, University of Miami School of Medicine, Miami, Florida, United States of America; 3 Department of Ophthalmology, Zhongshan Hospital of Fudan University, Shanghai, China; 4 School of Life Sciences, University of Nevada Las Vegas, Las Vegas, Nevada, United States of America; 5 Dept. of Molecular & Human Genetics, Baylor College of Medicine, Houston, Texas, United States of America; University of Bari Medical School, ITALY

## Abstract

Hepatoma-derived growth factor-related protein-3 (Hdgfrp3 or HRP-3) was recently reported as a neurotrophic factor and is upregulated in hepatocellular carcinoma to promote cancer cell survival. Here we identified HRP-3 as a new endothelial ligand and characterized its *in vitro* and *in vivo* functional roles and molecular signaling. We combined open reading frame phage display with multi-round *in vivo* binding selection to enrich retinal endothelial ligands, which were systematically identified by next generation DNA sequencing. One of the identified endothelial ligands was HRP-3. HRP-3 expression in the retina and brain was characterized by Western blot and immunohistochemistry. Cell proliferation assay showed that HRP-3 stimulated the growth of human umbilical vein endothelial cells (HUVECs). HRP-3 induced tube formation of HUVECs in culture. Wound healing assay indicated that HRP-3 promoted endothelial cell migration. HRP-3 was further confirmed for its *in vitro* angiogenic activity by spheroid sprouting assay. HRP-3 extrinsically activated the extracellular-signal-regulated kinase ½ (ERK1/2) pathway in endothelial cells. The angiogenic activity of HRP-3 was independently verified by mouse cornea pocket assay. Furthermore, *in vivo* Matrigel plug assay corroborated HRP-3 activity to promote new blood vessel formation. These results demonstrated that HRP-3 is a novel angiogenic factor.

## Introduction

Angiogenic factors regulate retinal blood vessels in physiological and pathological conditions. For example, vascular endothelial growth factor (VEGF) plays an important role in the pathogenesis of retinal vascular diseases, including exudative (i.e., vascular) age-related macular degeneration (AMD), diabetic macular edema (DME), proliferative diabetic retinopathy and retinopathy of prematurity [1]. Anti-VEGF therapies have been approved for DME and exudative AMD [2]. Identification of new angiogenic factors will delineate additional angiogenic mechanisms and help understand how retinal angiogenesis is regulated in various conditions.

Hepatoma-derived growth factor-related protein-3 (Hdgfrp3 or HRP-3) belongs to the family of hepatoma-derived growth factor (HDGF) family, which is composed of HDGF, HRP1-4 and lens epithelium-derived growth factor [3]. All the family members share a conserved N-terminal HATH domain. HRP-3 was recently characterized as a neurotrophic factor [4]. Purified recombinant HRP-3 promoted neuronal survival and neurite outgrowth. HRP-3 without a classical signal peptide can be secreted from cultured neurons and detected in culture medium. Antibody-mediated neutralization of extracellular HRP-3 resulted in neuronal degeneration [4]. HRP-3 is expressed only in the brain but not in other non-neuronal tissues [5]. Although the retina is a part of the central nervous system (CNS), HRP-3 expression in the retina has not been characterized. HRP-3 expression was highly upregulated in hepatocellular carcinomas to promote cancer cell survival [6]. However, HRP-3 has never been reported as an endothelial growth factor.

As a founding member of the family, HDGF has been extensively characterized at molecular and functional levels. The knowledge in HDGF may serve as a guide for HRP-3. HDGF without a N-terminal signal peptide was originally purified and identified from the conditioned media of the human hepatoma cell line Huh-7 and was capable of stimulating the proliferation of mouse 3T3 fibroblast cells [7,8]. In contrast to the restricted neuronal expression of HRP-3, HDGF is expressed in all tissues examined in a previous study, except the intestine, with the highest expression in the brain, testis and lung [5]. Upregulation of HDGF was reported in various cancers [9–13]. HDGF was described as a mitogenic factor for various types of cells. It stimulated the growth of endothelial cells, vascular smooth muscle cells, fibroblasts and hepatoma cells [14–17]. HDGF was reported as a neurotrophic factor [18].

Here we systematically identified endothelial ligands by open reading frame phage display (OPD) coupled with multi-round *in vivo* selection to enrich endothelial binding clones. All enriched ligands were subsequently identified by next generation DNA sequencing (NGS). One of the ligands identified by OPD-NGS was HRP-3 with high binding activity to retinal endothelium. We investigated HRP-3 as an angiogenic factor with a series of *in vitro* and *in vivo* functional characterizations. These results in turn support the validity of OPD-NGS technique for unbiased identification of endothelial ligands. The broad applicability of the new approach in vascular research is discussed.

## Materials and Methods

### Cell culture

Human umbilical vein endothelial cells (HUVECs) and human aorta endothelial cells (HAECs) were purchased from Lonza (Allendale, NJ) and cultured in complete EGM-2 medium (Lonza). The cells were used for experiments at Passage 4–8.

### *In vivo* binding selection

Two OPD cDNA libraries generated from mouse embryos at day 18 and mouse adult eyes were previously described [19,20]. Both libraries were amplified, purified according to Novagen T7Select System Manual (Millipore, Billerica, MA) with modifications. Briefly, BLT5615 bacteria were cultured in LB medium to OD_600_ at 0.5 and induced by IPTG (isopropyl β-D-1-thiogalactopyranoside, 1mM) at 37°C for 30 min with shaking [19]. The libraries (~4 x 10^9^ plaque form unit (pfu)) were added to BLT5615 (200 ml) and incubated with shaking at 37°C until bacterial lysis, followed by incubation with DNase I (40 μg, Sigma) for additional 15 min. After adding and dissolving NaCl (5 g), the lysates were centrifuged at 13,200 x g for 10 min at 4°C. Polyethylene glycol 8000 (PEG-8000, 20 g, Sigma) was added and dissolved in the supernatants, which were incubated at 4°C overnight and centrifuged under the same condition. The phage pellets were resuspended in a Tris-NaCl buffer (1 M NaCl, 10 mM Tris-HCl, pH8.0, 1 mM EDTA) and centrifuged. The supernatants were laid on a discontinuous CsCl gradient (20.8%, 31.25%, 41.7% and 62.5%) and centrifuged in Beckman SW41 rotor at 35,000 rpm for 60 min at 23°C. A phage band right above 41.7% CsCl was collected, dialyzed against PBS and titrated by phage plaque assay [19,20]. Two libraries were pooled together in equal titer to increase library representation.

C57BL/6 mice (22 weeks old) were anesthetized by intraperitoneal injection of ketamine (90 μg/g) and xylazine (8 μg/g). Purified libraries were intravenously injected into anesthetized mice (3 mice/round, 1 x 10^12^ pfu/mouse) and circulated for 20 min [21]. We reasoned that this should be sufficient time to allow the binding of displayed ligands to endothelial receptors, but not enough time for phages to get out of blood vessels with binding to non-endothelial cells. Endothelial binding phages may be minimally internalized through receptor-mediated mechanisms within such a short period of time. Even internalized, phages should not be degraded in endosomes within 20 min, as reported in our other studies [22]. These speculations were supported by the identification and independent validation of HRP-3 as an endothelial ligand in this paper. After 20-min circulation, unbound phages were removed by intracardial perfusion with PBS. The retinas were isolated and homogenized in PBS containing 1% Triton X-100 to release endothelium-bound phages, which were amplified in the bacteria and repurified by CsCl centrifugation as above. Purified phages were used as input for the next round of *in vivo* selection. After 3 rounds of selection, the cDNA inserts of enriched phage clones were amplified by PCR with primer 5’-GCGGTTCAGGCTCGCGGCCG-3’ and 5’-CCGCTCCCACTACCCTCGAG-3’. PCR products between 400–1,500 bp were purified from agarose gel and identified by NGS. NGS data were aligned against NCBI CCDS database to identify enriched ligands.

All animal procedures in this study were approved by the Institutional Animal Care and use Committee at the University of Miami (Protocol #13–229) and complied with the *Guide for the Care and Use of Laboratory Animals* published by the United States National Institutes of Health (NIH).

### Recombinant proteins

The coding sequences of mouse HRP-3 and HDGF were amplified with a C-terminal polyhistidine tag by PCR and cloned into pET-22b vector (Millipore) without the pelB leader sequence to generate HRP-3-polyhis and HDGF-polyhis plasmids. Recombinant HRP-3 and HDGF were expressed in BL21(DE3) bacteria at 16°C for 48 h, purified using cobalt columns, dialyzed against PBS and analyzed by SDS-PAGE, as described [23]. Glutathione S-transferase (GST)-polyhistindine was constructed, expressed and purified in a similar manner and showed no angiogenic activity in *in vitro* and *in vivo* assays (data not shown).

### Immunohistochemistry and Western blot

Anesthetized mice (C57BL/6, 6 weeks old) were intracardially perfused with 10% formalin. After euthanasia, the eyes were isolated and fixed overnight at 4°C. After removal of the cornea and lens, eye cups were incubated with sucrose gradient solutions (10% and 20% for 3 h each; 30% for overnight) at 4°C, followed by 3 rounds of freeze-thaw and OCT embedding. Frozen tissue sections in 7-μm thickness were incubated with rabbit anti-HRP-3 (Santa Cruz Biotechnology, Dallas, TX), followed by FITC-labeled goat anti-rabbit IgG antibodies. The nuclei were visualized with DAPI. The signals were analyzed by confocal microscopy.

Alternatively, mice were directly euthanized by CO_2_ inhalation, followed by cervical dislocation. The retinas were isolated, homogenized in RIPA buffer (Thermo Fisher Scientific, Waltham, MA), analyzed by Western blot using anti-HRP-3 antibody or anti-β-actin antibody. Total brain was isolated from the same mice, homogenized and used as a positive control.

### Proliferation assay

HUVECs and HAECs were seeded in 48-well plates in complete EGM-2 medium at 1 x 10^4^ cells/well with HRP-3, HDGF or VEGF-165 (Abcam, Cambridge, MA) at indicated concentrations. Fresh medium and growth factors were added every 24 h. Cells in each well were collected by trypsin digestion and quantified at 24 or 48 h in PBS with 1 mM trypan blue using a hemocytometer.

### Wound healing assay

HUVEC migration was analyzed by *in vitro* wound healing assay as described [24]. Briefly, HUVECs were cultured in 12-well plates until ~90–100% confluence. Cells were starved for 3 h in EBM-2 medium (Lonza) supplemented with 0.2% fetal bovine serum (FBS). A sterile 200-μl tip was used to create a defined and clear scratch approximate 1 mm in width in each well. The dislodged cells were immediately removed by rinsing, and the remaining cells were supplemented with fresh EBM-2 medium containing 0.2% FBS in the presence of HRP-3, VEGF or PBS. The migration of cells was monitored at 0 and 20 h by phase-contrast microscopy. After staining with calcein AM at 20 h, at least 6 images in each well were analyzed by fluorescence microscopy. The percentage of the denuded area covered by migrated cells within the original scratch was quantified using ImageJ software (NIH).

### Tube formation assay

The assay was performed with HUVECs as described with modifications [25]. Corning high concentration Matrigel (growth factor reduced) (Tewksbury, MA) was diluted 1:4 (vol/vol) in EBM-2 medium, plated in 96-well plates (50 μl/well) and allowed to solidify at 37°C for 30 min. HUVECs were starved overnight in serum-free EBM-2 medium and plated on the Matrigel (15,000 cells/well) in the presence of HRP-3, VEGF or PBS. Bright field images were obtained after 3 h of incubation at 37°C. Total tube length, number of branching points and number of the tubes per viewing field were quantified by ImageJ.

### Endothelial spheroid sprouting assay

The assay was performed with HUVECs as described [26]. Briefly, methocel solution was prepared by dissolving methycellulose (Sigma, St. Louis, MO) in EBM-2 medium at 1.2% and centrifuged at 5,000 x g for 2 h at 4°C to clear debris. HUVECs at 80% confluence were harvested, counted, resuspended in EBM-2 medium containing 20% methocel and 10% FBS, seeded at 750 cells/well in non-adhesive 96-well round-bottomed plates and cultured for 24 h. The spheroids were harvested, resuspended in EBM-2 medium containing fibrinogen (2.5 mg/ml) and aprotinin (0.05 mg/ml), and seeded in 24-well plates (~50 spheroids/ml/well). Clotting was induced by adding thrombin (12 units/ml) to each well. The spheroid-embedded fibrin gel was allowed to clot for 5 min at room temperature and then 20 min at 37°C. The fibrin gel was equilibrated with 1 ml of EBM-2 medium containing aprotinin (0.05 mg/ml) in the presence of HRP-3, VEGF or PBS, and incubated for 48 h at 37°C. Photographs were taken using a phase contrast microscope, and average sprout lengths were quantified using by ImageJ as described in S1 Fig.

### Corneal pocket assay

The assay was carried out as described with modifications [27]. Briefly, sterilized Whatman filter paper (Grade 3) (GE Healthcare Bio-Sciences, Piscataway, NJ) was cut into pieces (0.125 mm^2^/piece). The papers were soaked in the solution of HRP-3, VEGF or PBS for 2 h at 4°C, and implanted into corneal pockets in anesthetized C57BL/6 mice (8–10 weeks old; 1 paper/cornea; 2 pockets/mouse). After 6 days, angiogenesis in each eye was evaluated using a slit-lamp microscope and photographed. The number of new sprouting vessels into the cornea and their branching points were quantified. In addition, we modified a previous scoring system [28] to semiquantitatively analyze the number, density, length of visible corneal blood vessels (S1 Table). The mice were then euthanized by CO_2_, and immediately perfused intracardially with lipophilic fluorescent DiI dye [29]. The eyes were removed and fixed in 10% formalin for 24 h at 4°C. The corneas were dissected right at the limbus, flat-mounted in 50% glycerol/PBS, and imaged by confocal microscopy to detect DiI-labeled blood vessels.

### Matrigel plug assay

The assay was performed as described [30]. The high concentration Matrigel was diluted 1:1 in EBM-2 and mixed with heparin (320 μg/ml) in the presence of HRP-3, VEGF or PBS. The Matrigel was injected into anesthetized C57BL/6 mice (~10 week old) subcutaneously around the flank areas (500 μl/site, 2 sites/mouse). Mice were euthanized by CO_2_ at day 7, followed by cervical dislocation. The Matrigel plugs were removed, photographed, weighed, homogenized and centrifuged at 16,000 g for 15 min at 4°C. Hemoglobin content was quantified by directly measuring the supernatants at OD_405_, calculated against a standard curve generated with purified porcine hemoglobin (Sigma), and normalized against wet weight of the plug.

### ERK activation

ERK activation was detected as described with modifications [6]. Briefly, HUVECs or HAECs were seeded in 6-well plates precoated with gelatin and cultured to ~90% confluence. Cells were preincubated in 293 SFM II medium (Life Technologies, Grand Island, NY) for 15 min x 3 times at 37°C to reduce the effect of other growth factors. Then the cells were incubated with HRP-3 for 10 min in 37°C and lysed in RIPA buffer (Pierce Biotechnology, Rockford, IL) containing phosphatase inhibitor cocktail (Roche). The lysates were analyzed by Western blot using antibody against phosphorylated ERK1/2 (pEKR1/2), ERK1/2 or β-actin (Cell Signaling, Danvers, MA), followed by horseradish peroxidase-labeled secondary antibody for chemiluminescence to detect the signals. Epidermal growth factor (EGF, Life Technologies) has been used in many previous studies of ERK1/2 activation and was included as a positive control.

### Statistical analysis

Data were expressed as means ± s.e.m. and analyzed by one-way ANOVA test. Data were considered significant when *P* < 0.05.

## Results

### Identification of HRP-3 as a new endothelial ligand

Endothelial ligands are traditionally identified on a case-by-case basis with technical challenges. We recently developed OPD as a new technology of functional proteomics to identify endogenous proteins with specific binding or functional activity [19,31,32]. One of the applications was unbiased identification of phagocytosis ligands [22,33]. In this study, we used OPD for systematic identification of endothelial ligands.

**Fig 1:**
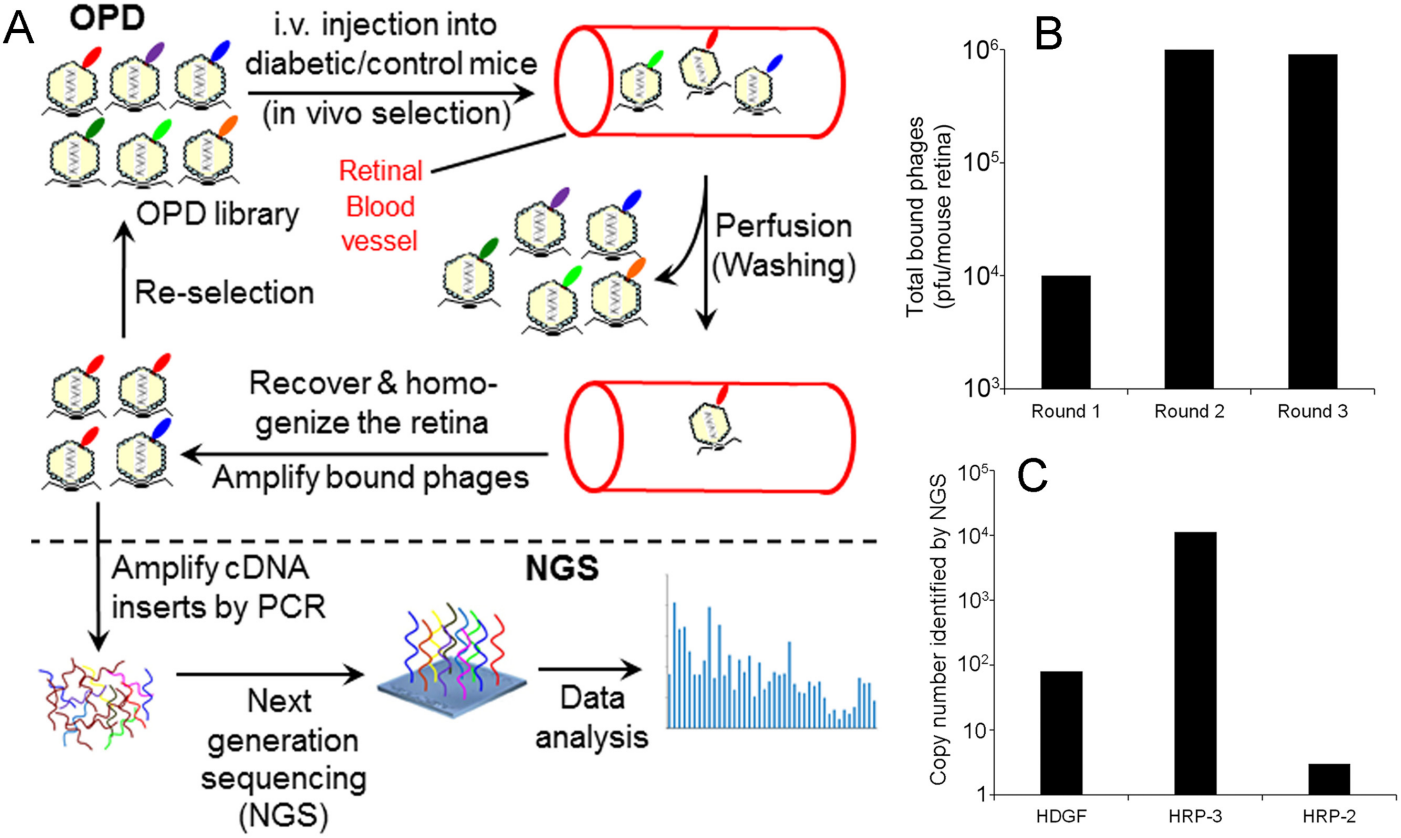
Identification of HRP-3 as an endothelial ligand. (A) OPD-based *in vivo* binding selection. OPD cDNA libraries were purified and intravenously injected into mice. After circulating for 20 min, unbound phages were removed by intracardial perfusion. The retinas were isolated and homogenized to release endothelium-bound phages, which were amplified in bacteria and used as in put for the next round of selection. After 3 rounds of selection, the cDNA inserts of enriched phage clones were amplified and identified by NGS with simultaneous quantification of the copy numbers of cDNA inserts for individual clones. (B) Total phages bound to retinal endothelium. At the end of each round of selection, total endothelium-bound phages in the retinal homogenate were quantified by plaque assay and expressed as plaque forming units (pfu)/retina. (C) The copy number of the cDNA inserts for HDGF, HRP-2 and HRP-3 quantified by NGS.

We performed *in vivo* binding selection with OPD cDNA libraries (Fig 1A, top panel). Three rounds of selection resulted in 90-fold increase in total phage binding activity to retinal endothelium (Fig 1B). Instead of manually isolating and identifying individual enriched phage clones, we amplified the cDNA inserts of all enriched phages and sequenced them by NGS (Fig 1A, bottom panel). All identified sequences were aligned against NCBI CCDS database to identify enriched endothelial ligands. A total of 489,126 valid sequences were identified and matched to the database. Among identified putative ligands was HRP-3 with 11,140 copies of its cDNA insert detected by NGS (Fig 1C). In addition, HDGF and HRP-2 were identified with 80 and 3 copies of their cDNA inserts, respectively. No other HDGF family member was detected by OPD-NGS. These data indicated that HRP-3 had the highest binding activity to retinal endothelium among the three identified family members, implicating that HRP-3 may play an important role in endothelial regulation.

### HRP-3 expression in the brain and retina

**Fig 2:**
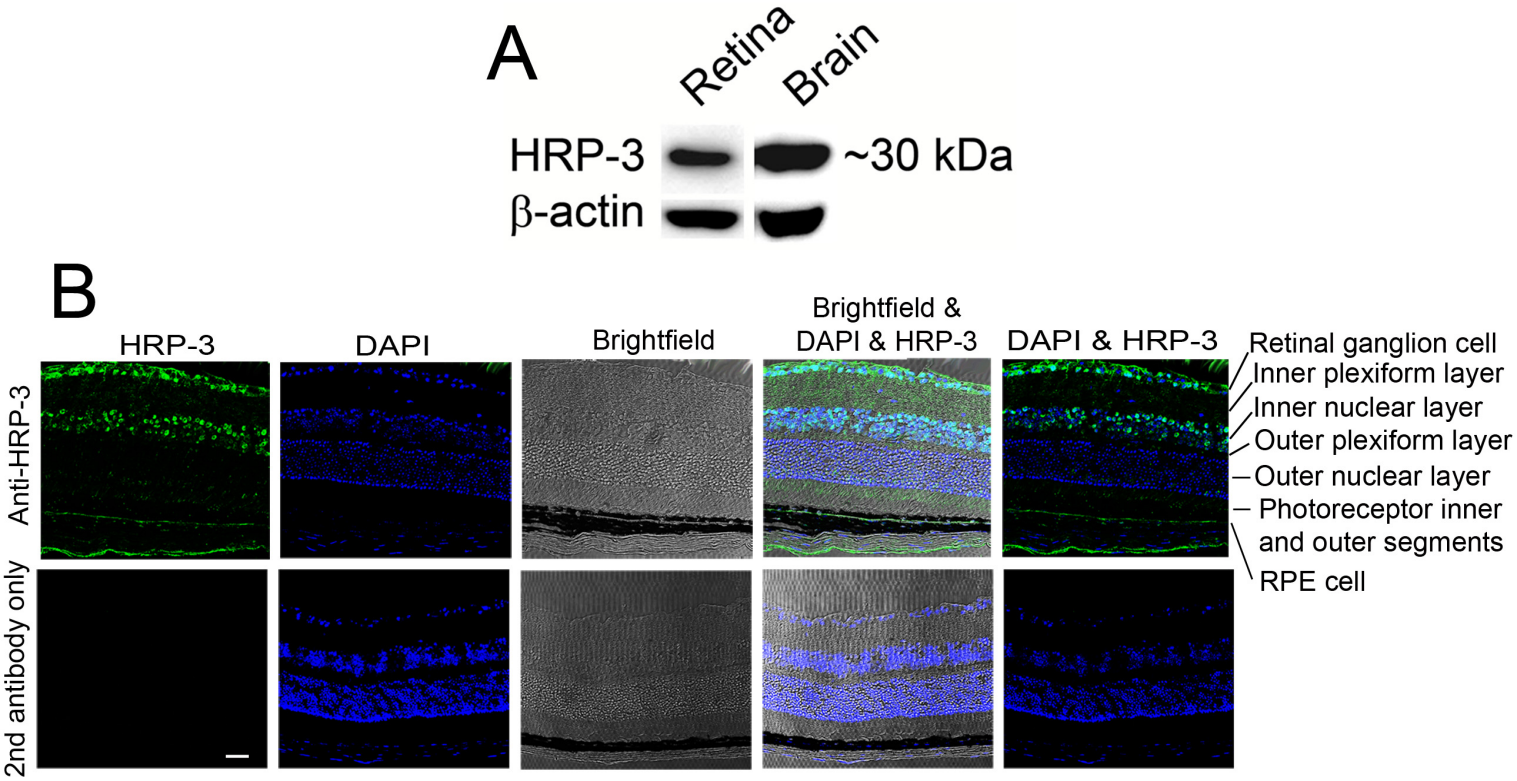
HRP-3 expression in the retina. (A) Western blot to detect HRP-3 expression in the retina. Brain homogenate was included as a positive control. (B) Immunohistochemical analysis of HRP-3 expression in the retina. HRP-3 is predominantly expressed in the retinal ganglion cell layer and inner nuclear layer. HRP-3 is also expressed in retinal pigment epithelial (RPE) cells at a relatively low level. No HRP-3 was detected in photoreceptors, including outer nuclear layer, photoreceptor inner and outer segments (OS). Scale bar = 50 μm. These results were validated three times with similar outcomes.

Because our *in vivo* binding selection was performed in mouse retina in Fig 1, it is important to investigate the expression of HRP-3 in the retina. Our Western blot analysis detected HRP-3 expression in both the brain and retina (Fig 2A). Immunohistochemistry revealed that HRP-3 is mainly expressed in the ganglion cells and inner nuclear layer (Fig 2B). Minor expression of HRP-3 was also detected in retinal pigment epithelial (RPE) cells. Few signals were detected in other layers of the retina, including inner and outer plexiform layers, outer nuclear layer, and photoreceptor inner or outer segments. No signal was detected in the absence of the primary antibody.

### HRP-3 is an endothelial growth factor

**Fig 3:**
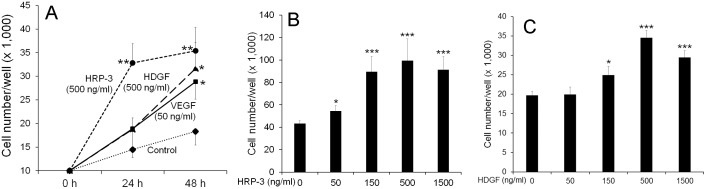
HRP-3 promotes endothelial cell growth. (A) Activity of HRP-3, HDGF and VEGF to stimulate HUVEC growth at different times. (B) Dose-dependent activity of HRP-3 to induce HUVEC growth at 48 h. (C) Dose-dependent activity of HDGF to facilitate HUVEC growth at 48 h. Data are mean ± s.e.m. in one representative experiment. n = 4 (4 wells/group). These results were validated three times with similar outcomes. *P<0.05, **P<0.01, ***P<0.001, vs. control.

Given that HDGF was previously reported as an endothelial mitogen [14], we characterized the activity of HRP-3 to stimulate the proliferation of endothelial cells. We expressed HRP-3 and HDGF with a C-terminal polyhistidine tag in bacteria and purified the recombinant proteins (S2 Fig). Purified proteins were analyzed for their abilities to stimulate the proliferation of HUVECs and HAECs. The results showed that HRP-3 at 500 ng/ml significantly induced HUVEC proliferation at 24 and 48 h (*P*<0.01) (Fig 3A). In contrast, HDGF (500 ng/ml) and VEGF (50 ng/ml) stimulated significant endothelial proliferation only at 48 h (*P*<0.05). Dose curve study showed that HRP-3 at 50, 150, 500 and 1,500 ng/ml significantly induced endothelial cell proliferation at 48 h (Fig 3B and 3C). Similarly, HDGF at 150, 500 and 1,500 significantly promoted endothelial cell growth (Fig 3D). HRP-3 was also capable of stimulating the proliferation of HAECs (S3 Fig). These results suggest that HRP-3 is an endothelial mitogen.

### HRP-3 induces tube formation

**Fig 4:**
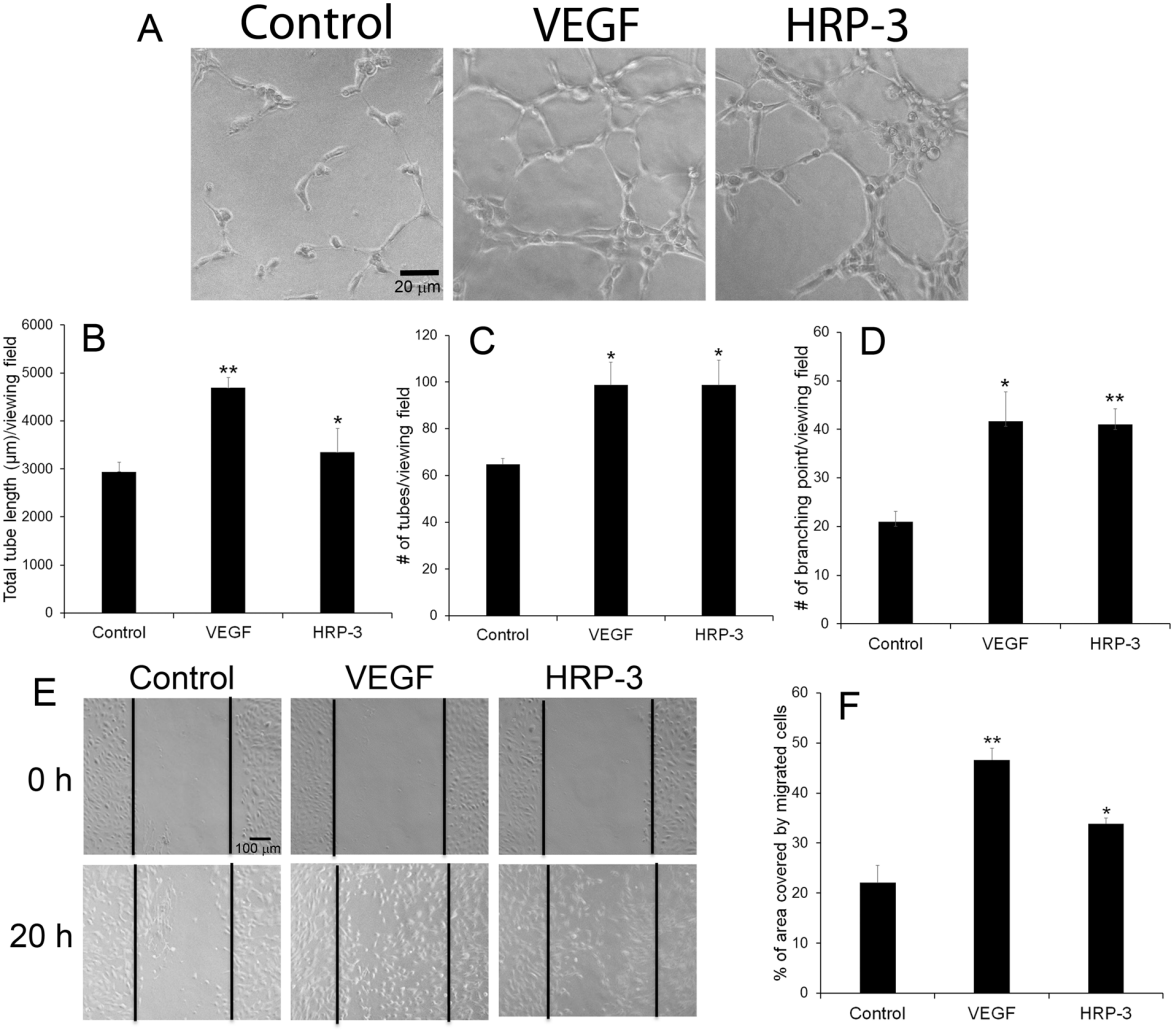
HRP-3 induces endothelial tube formation and migration. (A) Representative images of the tube formation. HUVECs were starved overnight, plated on Matrigel gel and cultured in serum-free EBM-2 medium in the presence HRP-3 (200 ng/ml), VEGF (50 ng/ml) or PBS for 3 h. Bar = 20 μm. (B-D) Quantification of the tube formation. Total tube length (B), number of tubes (C) and number of branching points (D) in each viewing field was quantified and compared (n = 3 viewing fields). (E) Representative images of endothelial migration by wound healing assay. HUVECs were cultured in 12-well plates to nearly confluence. A scratch was created in each well. HRP-3 (500 ng/ml), VEGF (50 ng/ml) or PBS was incubated with the cells for 20 h. Bar = 100 μm. (F) The percentage of the denuded area covered by migrated cells within the original scratch was quantified (n = 3 wells). These results of tube formation and migration were validated three times with similar outcomes. Data are mean ± s.e.m. *P<0.05, **P<0.01, vs. control.

*In vitro* Matrigel assay was performed to investigate the effect of HRP-3 on endothelial cell tube formation. Appropriate HRP-3 concentrations for functional assays described in this paper were predetermined by simplified pilot dose studies similar to Fig 3B (not shown). The results revealed that HRP-3 promoted the tube formation of HUVECs at 3 h (Fig 4A). As a positive control, VEGF showed a similar effect. Quantification of the tube length, number of branching points and number of the tubes in different groups indicated that both HRP-3 and VEGF significantly induced the tube formation (Fig 4B–4D).

### HRP-3 stimulates endothelial cell migration

The migration of endothelial cells represents an important process of angiogenesis. Therefore, we investigated the capability of HRP-3 to induce endothelial cell migration by performing *in vitro* wound healing assay [14] to quantify the HUVECs migrated into the scratched area. The results showed that HRP-3 and VEGF significantly induced HUVEC migration (Fig 4E and 4F), suggesting that HRP-3 is likely an angiogenic factor.

### HRP-3 promotes endothelial cell sprouting

**Fig 5:**
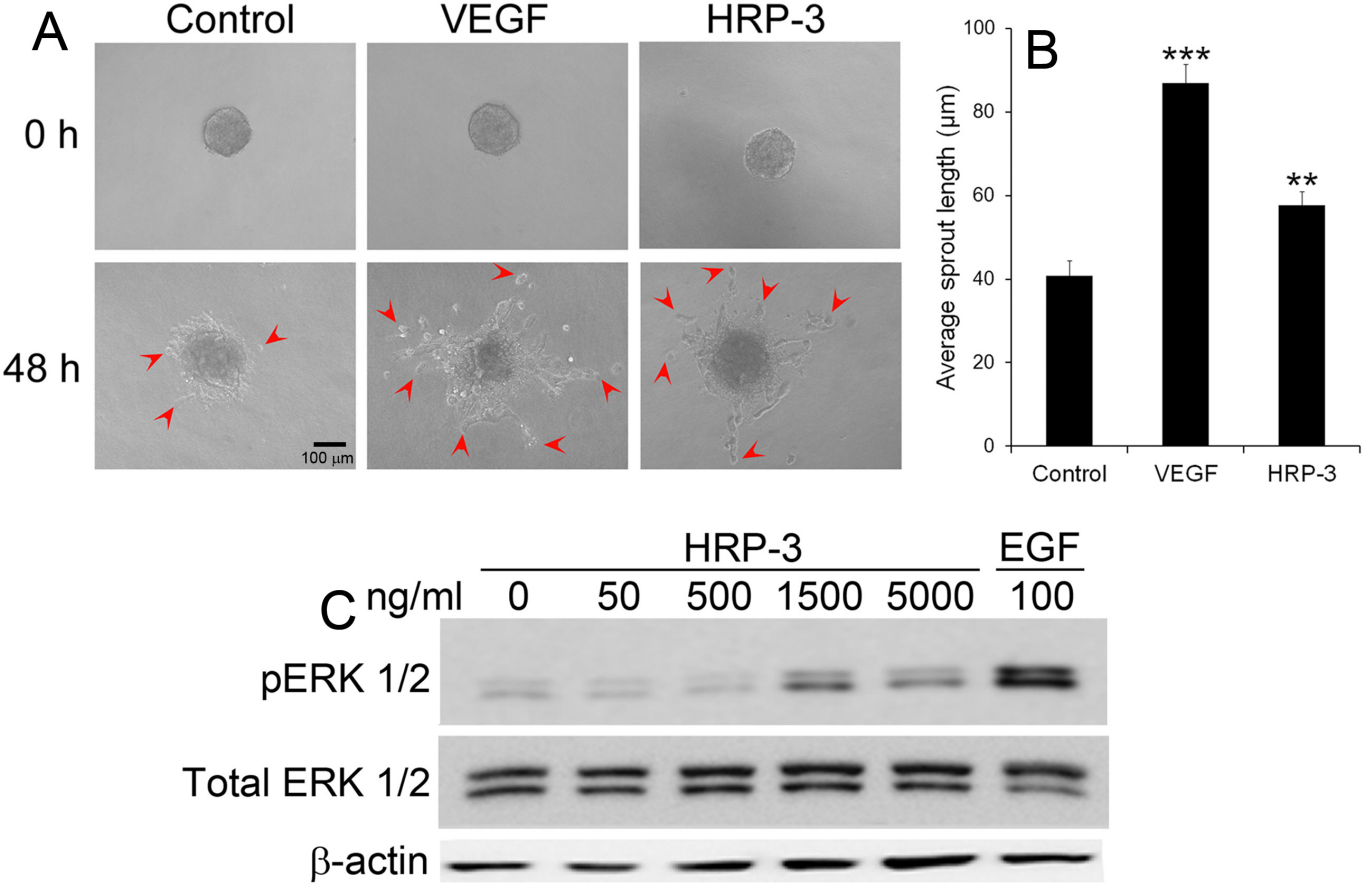
HRP-3 induces endothelial cell sprouting and ERK activation. (A) Endothelial cell sprouts induced by HRP-3. HUVEC spheroids were embedded in fibrin gel and cultured in EBM-2 medium in the presence of HRP-3 (10 ng/ml), VEGF (2.5 ng/ml) or PBS for 48 h. Bar = 100 μm. (B) Quantification of endothelial cell sprouts. The average length of sprouts per spheroid was quantified as described in S1 Fig. A total of 10 spheroids per group were quantified (n = 10). Data are mean ± s.e.m. in one representative experiment. **P<0.01, *** *P*<0.001, vs. control. (C) HRP-3 activates ERK pathway. HUVECs were starved in serum-free medium for 45 min, followed by incubation with HRP-3 or EGF (positive control) for 10 min. The cell lysate were analyzed by Western blot using antibody against ERK, phospho-ERK (pERK) or β-actin. Both experiments were validated three times with similar outcomes.

We further assessed the role of HRP-3 in a 3D model of endothelial morphogenesis *in vitro*. As shown in Fig 5A, HRP-3 induced the sprouting of HUVEC aggregates embedded in a fibrin gel. VEGF as a positive control had a similar effect on endothelial cell sprouting. Quantification of average length of the sprouts indicated that both HRP-3 and VEGF induced significant sprouting (Fig 5B).

### HRP-3 activates ERK

The mechanism by which HRP-3 regulates endothelial cells is not clear. HRP-3 was reported to extrinsically activate ERK pathway in hepatocellular carcinoma cells [6]. Given ERK as a major proliferation pathway that plays an important role in angiogenesis, we predict that HRP-3 activates this pathway in endothelial cells. To test this possibility, we starved HUVECs in serum-free medium, followed by 10-min incubation with HRP-3. A dose-dependent increase in ERK1/2 phosphorylation was observed in HUVECs treated with HRP-3 (Fig 5C). A similar ERK1/2 phosphorylation was induced by HRP-3 in HAECs (S4 Fig). EGF as a positive control induced ERK phosphorylation in both HUVECs and HAECs (Fig 5C, S4 Fig).

### HRP-3 is an angiogenic factor in vivo

**Fig 6:**
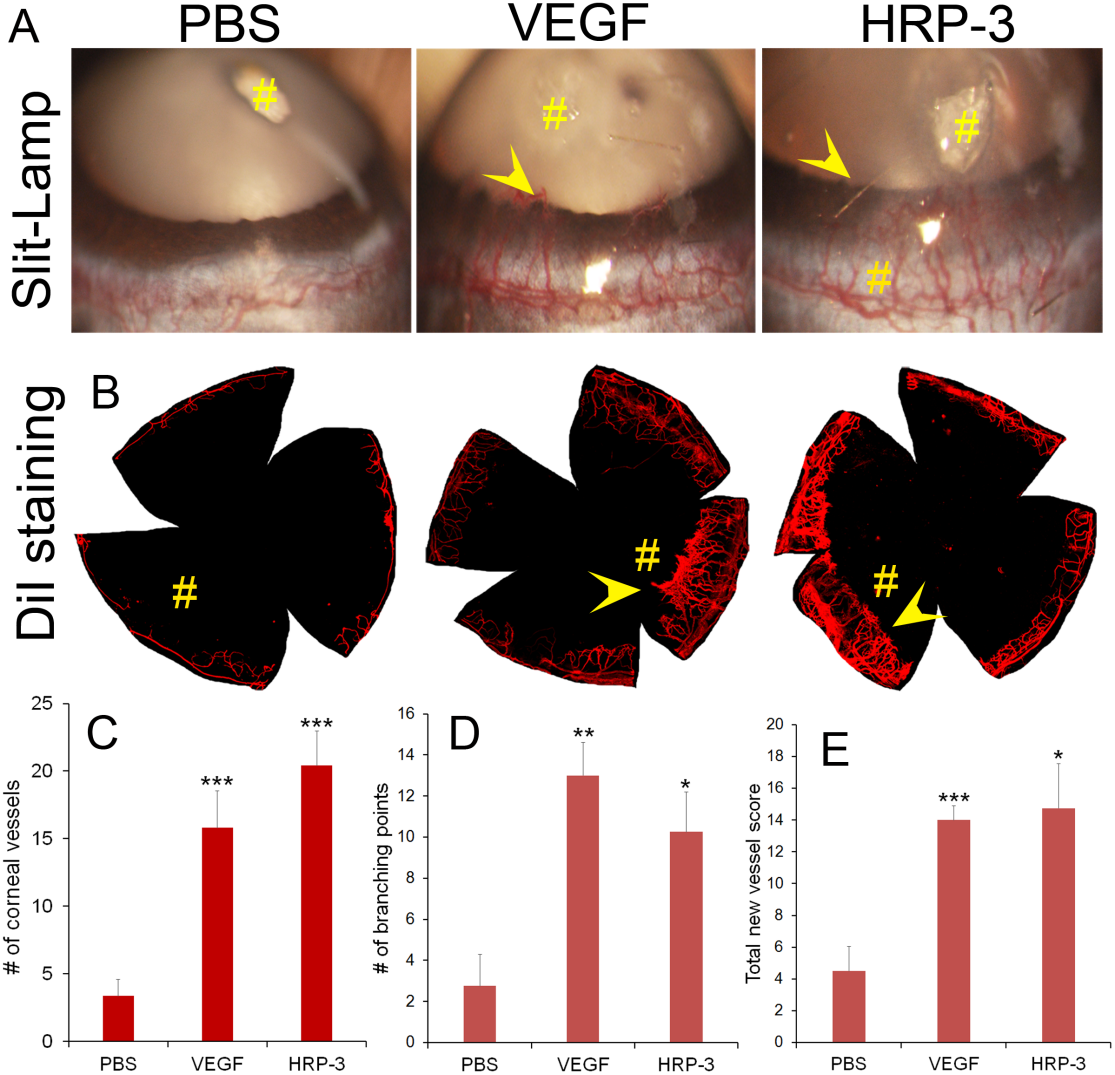
HRP-3 stimulates angiogenesis by corneal pocket assay. (A) Representative images of corneal angiogenesis. Small pieces of filter papers presoaked in HRP-3 (1 μg/μl), VEGF (100 ng/μl) or PBS were implanted in corneal pockets to induce vascular sprouting into the cornea for 6 days. # indicates filter paper position. Arrowheads indicate corneal blood vessels. (B) Representative images of corneal blood vessels labeled with fluorescent DiI dye. (C) The number of new sprouting vessels into the cornea was quantified and compared among HRP-3 (n = 5), VEGF (n = 5) and PBS (n = 10). (D) The number of branching points of corneal vessels and (E) total new vessel score were also quantified for the latest cases of HRP-3 (n = 4), VEGF (n = 4) and PBS (n = 5). Data are mean ± s.e.m. *P<0.05, **P<0.01, ***P<0.001, vs. control PBS.

Regardless of its angiogenic activities quantified by various *in vitro* assays, HRP-3 as an angiogenic factor should be independently verified *in vivo*. We characterized the angiogenic activity of HRP-3 *in vivo* by corneal pocket angiogenesis assay and Matrigel plug assay. The cornea is an avascular tissue with convenience to visualize and quantify neovascularization. For corneal pocket assay, small pieces of filter paper were presoaked in the solution of HRP-3, VEGF or PBS and implanted into mouse corneal pockets. After 6 days, corneal blood vessels were detected by slit-lamp microscopy and verified by staining with lipophilic fluorescent DiI dye (Fig 6A and 6B). Quantification of newly-formed corneal blood vessels and their branching points indicated that HRP-3 and VEGF significantly stimulated angiogenesis (Fig 6C and 6D). Moreover, a semiquantitative scoring method by combining the number, density and length of corneal blood vessels (S1 Table) showed that HRP-3 and VEGF induced significant corneal angiogenesis (Fig 6E).

**Fig 7:**
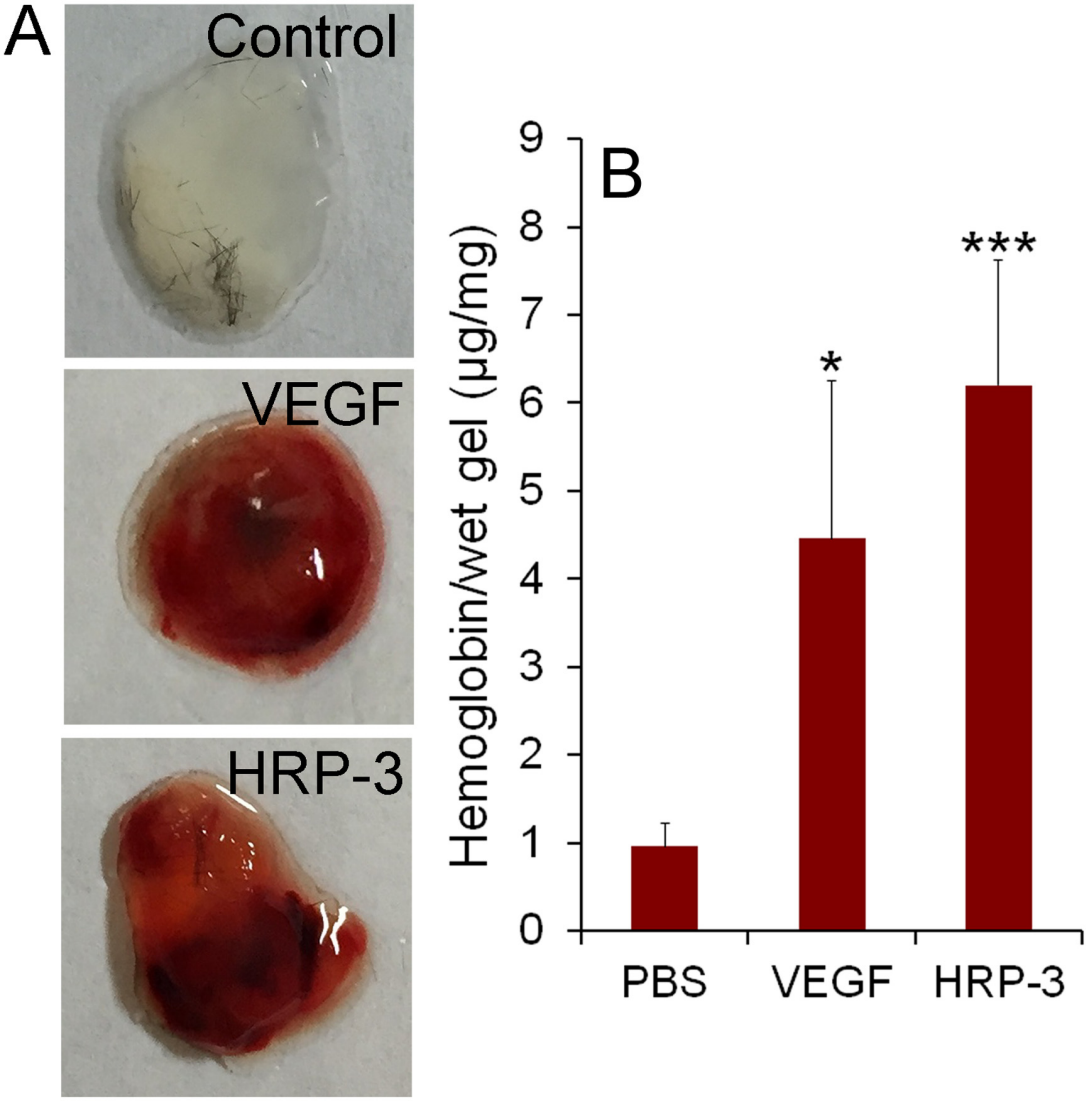
HRP-3 stimulates angiogenesis by Matrigel plug assay *in vivo*. C57BL/6 mice were injected s.c. with 0.5 ml of Matrigel containing HRP-3 (2 μg/ml), VEGF (250 ng/ml) or PBS around the flank areas. After 7 days, the mice were euthanized, and the Matrigel plugs were excised and photographed. (A) Representative images of Matrigel plugs. (B) Quantification of hemoglobin concentration in each Matrigel plug for HRP-3 (n = 5), VEGF (n = 6) or PBS (n = 11). Data are mean ± s.e.m. *P<0.05, ***P<0.001, vs. control PBS.

To further confirm HRP-3 as an angiogenic factor, we performed the second *in vivo* angiogenesis study by Matrigel plug assay in the presence of HRP-3, VEGF or PBS. The results showed that both HRP-3 and VEGF induced blood vessel growth into the Matrigel plugs (Fig 7A and 7B). Total blood vessel levels were quantified by measuring hemoglobin content in the Matrigel gel. Both HRP-3 and VEGF significantly stimulated the growth of blood vessels. Therefore, both *in vivo* studies indicated that HRP-3 is a novel angiogenic factor.

## Discussion

Compared to the well-characterized HDGF, HRP-3 is poorly studied. HRP-3 without a classical signal peptide was originally described as an intracellular protein with a nuclear localization signal [5]. The Franken lab reported that HRP-3 was expressed in the nuclei and neurites of neocortical neurons in culture [5,34]. The same group further demonstrated that HRP-3 is located in the cytoplasm and neurites of cortical neurons in mouse embryos but relocalizes continuously to the nuclei during the development [34]. Their data further suggest that HRP-3 expression in the neurites of immature neurons promotes neurite outgrowth by facilitating tubulin polymerization and microtubule stabilization. Our immunohistochemistry showed that the protein was detected predominantly in the ganglion cell layer and inner nuclear layer of adult mice (Fig 2). Our results are consistent with their finding that HRP-3 is exclusively expressed in the nuclei of adult neurons [34]. Surprisingly, photoreceptor cells as specialized sensory neurons do not express HRP-3.

Despite the lack of a classical signal peptide, a recent study characterized HRP-3 as a neurotrophic factor that was secreted from cultured neurons to extrinsically stimulate neuronal survival and promote neurite outgrowth [4]. The unconventional secretion of HRP-3 is consistent with HDGF, which was originally identified and purified from the conditioned media of Huh-7 cell line [7,8]. However, the molecular mechanisms of the unconventional secretion for both proteins are unclear. A previous study indicated that the unconventional section of HDGF requires the N-terminal 10 amino acids and that phosphorylation of serine 165 in its C-terminal region plays a critical role in the secretion process [35]. HRP-3 and HDGF share 78% identity in the N-terminal 100 amino acid residues of the PWWP/HATH domain and only 39% identity in the C-terminal non-HATH region. It is likely that the HATH domain of HRP-3 may also involve in its unconventional secretion.

Cytoplasmic HRP-3 interacts with microtubules and promotes neurite outgrowth [34]. HRP-3 is upregulated in hepatocellular carcinoma (HCC) and is required for anchorage-independent survival and chemoresistance of HCC [6]. Knockdown of HRP-3 failed to affect anchorage-dependent growth of HCC cells. Moreover, HRP-3 specifically activated ERK pathway in HCC [6]. HRP-3 silencing induced apoptosis of H1299 lung epithelial carcinoma cells via reactive oxygen species (ROS)-dependent and p53-independent NF-κB activation [36]. Deletion of HRP-3 resulted in apoptotic sensitization of radioresistant A549 lung epithelial carcinoma cells via ROS-dependent p53 activation [37].

While HDGF has been reported as an endothelial mitogen with angiogenic activity [14,38,39], HRP-3 has never been described as an endothelial ligand. In this study we identified HRP-3 as an endothelial-binding protein by OPD-NGS and provided multiple lines of evidence, both *in vitro* and *in vivo*, to demonstrate that HRP-3 is a new angiogenic factor. HRP-3 expressed in neurons may be secreted to regulate endothelial cells in a paracrine manner. Since the surface receptor(s) of HRP-3 and HDGF remains elusive, it is unclear if these two ligands share the same receptor(s). The data in Fig 1C implicate that HRP-3 may have much higher binding affinity than HDGF if they share the same receptor. Otherwise, the relatively high binding activity in Fig 1C implicates that HRP-3 receptor either is expressed at a higher level on the endothelium or has a higher affinity than HDGF receptor. The speculations are yet to be validated by the identification of their receptors on endothelial cells.

HDGF is the prototype for the family and the most investigated member. HDGF was originally identified as secretory heparin-binding protein [7,8]. Extracellular HDGF can be internalized through the binding of HATH 81–100 amino acids (HATH_81–100_) to cell surface heparan sulfate [40]. Intracellular HDGF with a nuclear signal can be transported into the nucleus [15]. HDGF harbors two mitogenic domains: one in the C-terminal non-HATH region responsible for intranuclear stimulation of DNA synthesis [17] and the other in the HATH_81–100_ region exerting its mitogenic activity from the cell surface [41]. Mutation K96A in the HATH domain abolishes HDGF binding to the cell surface and its mitogenic activity but does not affect its internalization activity [40,41]. These results suggest that HDGF domain recognizing its cell surface receptor for cell proliferation is different from heparan sulfate-binding domain to mediate HDGF internalization. Given the sequence homology in their HATH domains, HRP-3 may stimulate endothelial proliferation and angiogenesis through the similar mechanisms.

A unique approach of this study is to systematically identify endothelial ligands by OPD-NGS. Phage display with antibody libraries or random peptide libraries has been widely used to identify endothelial binding antibodies or peptides by *in vivo* binding selection [21,42]. Although identified antibodies or unnatural peptides can be used for drug targeting [21,43] or vascular imaging [44], they are not VEGF-like endogenous ligands to help understand extrinsic regulation of endothelial cells. Similarly, phage display with conventional cDNA libraries of cellular proteins identifies a high percentage of out-of-frame unnatural short peptides, instead of endogenous ligands [31,32]. We recently developed OPD for unbiased identification of phagocytosis ligands by phagocytosis-based functional cloning (PFC) selection [22,33]. The validity of this new approach was demonstrated by independently characterizing identified phagocytosis ligands [22,45]. This study further expanded OPD to endothelial ligands by *in vivo* binding selection. The combination of OPD with NGS converted manual screening of individual enriched clones into high-throughput mapping of all enriched ligands in the absence of receptor information. In this regard, we propose that OPD-NGS is the first paradigm of “ligandomics” for global mapping of cell-wide ligands. Ligandomics has not been hitherto possible to any cells. We predict that OPD-NGS will drastically improve our technical capability to identify unknown endothelial ligands.

## Supporting Information

S1 Fig Method to quantify average length of sprouts. The spheroids in (A) and (B) are identical. In the sharp contrast, the core spheroid kept its original shape, even with the sprouts. (A) Eight longest lines are drawn for sprouts from the center. (B) Eight lines are drawn for the core spheroid. Average length of 8 lines in each panel was quantified by ImageJ. Average sprout length was calculated based on the formula for each spheroid. A total of 10 spheroids were quantified in each group.
(PDF)

S2 Fig Purified HRP-3 and HDGF. Recombinant HRP-3 and HDGF were expressed as bacteria with a C-terminal polyhistidine tag, purified using cobalt columns and analyzed by SDS-PAGE with Commassie blue staining.
(PDF)

S3 Fig HRP-3 enhances the growth of HAECs. The proliferation assay with HAECs was performed as in Fig 3. HRP-3 (500 ng/ml), VEGF (50 ng/ml) or PBS was incubated with HAECs for 48 h. Total number of cells in each well was quantified and compared (n = 8). Data are mean ± s.e.m., **P<0.01, vs. control.
(PDF)

S4 Fig HRP-3 activates ERK signaling pathway in HAECs. The experimental procedure was the same as to HUVECs in Fig 5C. Briefly, HAECs were incubated in serum-free medium for 15 min x 3 times, then incubated with HRP-3 or EGF for 10 min. Cells were lysed and analyzed by Western blot using antibodies against phospho-ERK (pERK), ERK or β-actin.
(PDF)

S1 Table Criteria for grading the severity of corneal neovascularization. (PDF)
