# Supplementary material for: Hepatoma-Derived Growth Factor-Related Protein-3 Is a Novel Angiogenic Factor
Source: PLoS One. 2015 May 21;10(5):e0127904. doi: 10.1371/journal.pone.0127904 (PMC4440747; doi:10.1371/journal.pone.0127904)
Supplement: S1 Fig — The spheroids in (A) and (B) are identical. In the sharp contrast, the core spheroid kept its original shape, even with the sprouts. (A) Eight longest lines are drawn for sprouts from the center. (B) Eight lines are drawn for the core spheroid. Average length of 8 lines in each panel was quantified by ImageJ. Average sprout length was calculated based on the formula for each spheroid. A total of 10 spheroids were quantified in each group. (PDF) [file pone.0127904.s001.pdf]

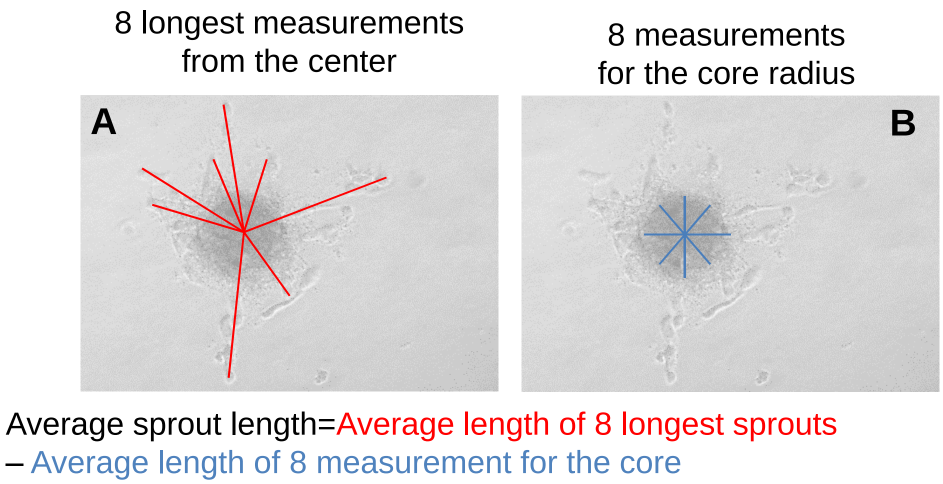

S1 Fig. Method to quantify average length of sprouts. The spheroids in (A) and (B) are identical. In the sharp contrast, the core spheroid kept its original shape, even with the sprouts. (A) Eight longest lines are drawn for sprouts from the center. (B) Eight lines are drawn for the core spheroid. Average length of 8 lines in each panel was quantified by ImageJ. Average sprout length was calculated based on the formula for each spheroid. A total of 10 spheroids were quantified in each group.

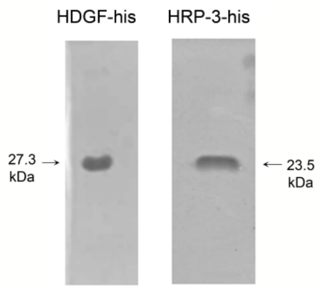

Fig. S2. **Purified HRP-3 and HDGF**. Recombinant HRP-3 and HDGF were expressed as bacteria with a C-terminal polyhistidine tag, purified using cobalt columns and analyzed by SDS-PAGE with Commassie blue staining.

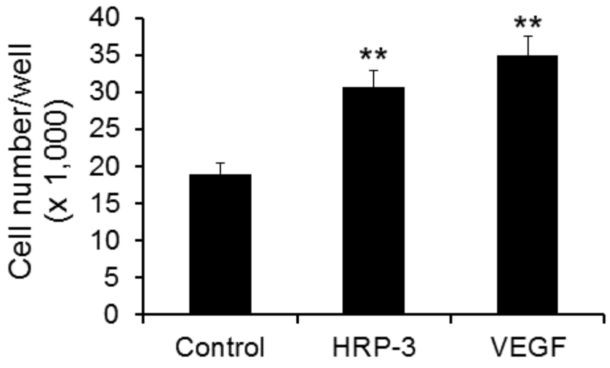

Fig. S3. **HRP-3 enhances the growth of HAECs**. The proliferation assay with HAECs was performed as in Figure 2. HRP-3 (500 ng/ml), VEGF (50 ng/ml) or PBS was incubated with HAECs for 48 h. Total number of cells in each well was quantified and compared (n=8). Data are mean ± s.e.m., t-test, ***P*<0.01, vs. control.

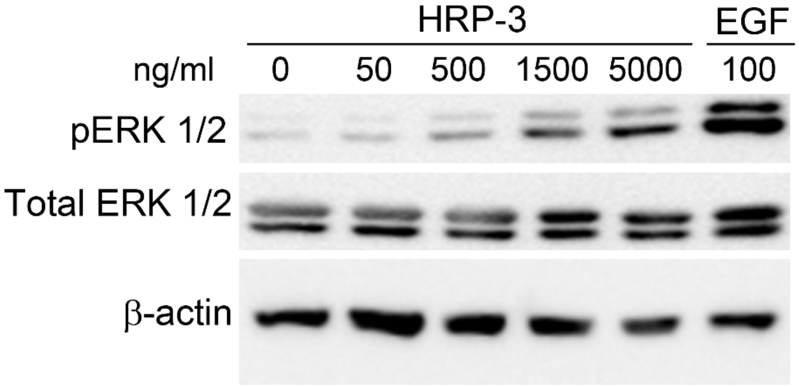

Fig. S4. **HRP-3 activates ERK signaling pathway in HAECs**. The experimental procedure was the same as to HUVECs in Figure 4C. Briefly, HAECs were incubated in serum-free medium for 15 min x 3 times, then incubated with HRP-3 or EGF for 10 min. Cells were lysed and analyzed by Western blot using antibodies against phospho-ERK (pERK), ERK or β-actin.
