# Supplementary material for: Hepatoma-Derived Growth Factor-Related Protein-3 Is a Novel Angiogenic Factor
Source: PLoS One. 2015 May 21;10(5):e0127904. doi: 10.1371/journal.pone.0127904 (PMC4440747; doi:10.1371/journal.pone.0127904)
Supplement: S2 Fig — Recombinant HRP-3 and HDGF were expressed as bacteria with a C-terminal polyhistidine tag, purified using cobalt columns and analyzed by SDS-PAGE with Commassie blue staining. (PDF) [file pone.0127904.s002.pdf]

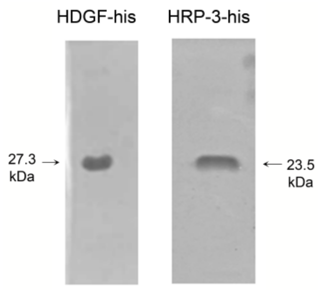

S2 Fig. Purified HRP-3 and HDGF. Recombinant HRP-3 and HDGF were expressed as bacteria with a C-terminal polyhistidine tag, purified using cobalt columns and analyzed by SDS-PAGE with Commassie blue staining.
