# Supplementary material for: Hepatoma-Derived Growth Factor-Related Protein-3 Is a Novel Angiogenic Factor
Source: PLoS One. 2015 May 21;10(5):e0127904. doi: 10.1371/journal.pone.0127904 (PMC4440747; doi:10.1371/journal.pone.0127904)
Supplement: S3 Fig — The proliferation assay with HAECs was performed as in Fig 3. HRP-3 (500 ng/ml), VEGF (50 ng/ml) or PBS was incubated with HAECs for 48 h. Total number of cells in each well was quantified and compared (n = 8). Data are mean ± s.e.m., **P<0.01, vs. control. (PDF) [file pone.0127904.s003.pdf]

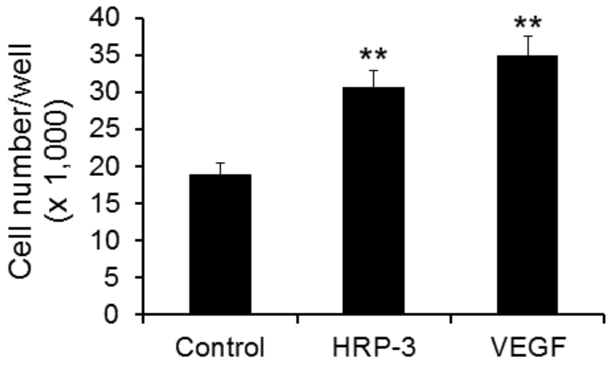

S3 Fig. HRP-3 enhances the growth of HAECs. The proliferation assay with HAECs was performed as in Figure 2. HRP-3 (500 ng/ml), VEGF (50 ng/ml) or PBS was incubated with HAECs for 48 h. Total number of cells in each well was quantified and compared (n=8). Data are mean ± s.e.m., t-test, $**P<0.01$, vs. control.
