# Supplementary material for: Hepatoma-Derived Growth Factor-Related Protein-3 Is a Novel Angiogenic Factor
Source: PLoS One. 2015 May 21;10(5):e0127904. doi: 10.1371/journal.pone.0127904 (PMC4440747; doi:10.1371/journal.pone.0127904)
Supplement: S4 Fig — The experimental procedure was the same as to HUVECs in Fig 5C. Briefly, HAECs were incubated in serum-free medium for 15 min x 3 times, then incubated with HRP-3 or EGF for 10 min. Cells were lysed and analyzed by Western blot using antibodies against phospho-ERK (pERK), ERK or β-actin. (PDF) [file pone.0127904.s004.pdf]

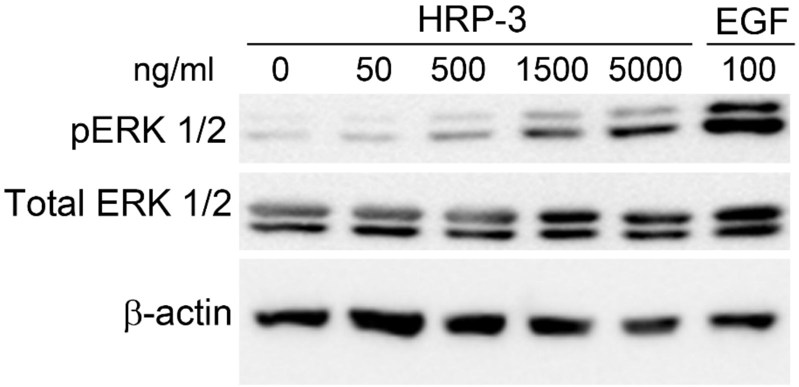

S4 Fig. HRP-3 activates ERK signaling pathway in HAECs. The experimental procedure is the same as to HUVECs in Fig. 4C. Briefly, HAECs were incubated in serum-free medium for 15 min x 3 times, then incubated with HRP-3 or EGF for 10 min. Cells were lysed and analyzed by Western blot using antibodies against phospho-ERK (pERK), ERK or β-actin.
