# Supplementary material for: Hepatoma-Derived Growth Factor-Related Protein-3 Is a Novel Angiogenic Factor
Source: PLoS One. 2015 May 21;10(5):e0127904. doi: 10.1371/journal.pone.0127904 (PMC4440747; doi:10.1371/journal.pone.0127904)
Supplement: S1 Table — (PDF) [file pone.0127904.s005.pdf]

S1 Table. Criteria for grading the severity of corneal neovascularization

Score	Number of Quadrants with corneal neovascularization	Number of corneal vessels / quadrant	Length of longest corneal vessel between limbus and cornea center / quadrant	Number of branch points / quadrant
1	1	1-5	0.05-0.25	1-5
2	2	6-10	0.26-0.50	6-10
3	3	11-15	0.51-0.75	11-15
4	4	>15	>0.75	16+

Note. Cornea neovascularization was assessed using a modified scoring system. A score of 0-4 was assigned for number, length and branch points for new cornea vessels, as indicated in the table above. The total score for each mouse was used as an indicator for corneal angiogenesis.
